# PI3K/Akt/mTOR信号转导通路与非小细胞肺癌

**DOI:** 10.3779/j.issn.1009-3419.2010.12.13

**Published:** 2010-12-20

**Affiliations:** 200233 上海，上海交通大学附属第六人民医院呼吸内科 Department of Respiratory Medicine, Shanghai Sixth People's Hospital Afliated to Shanghai Jiao Tong University, Shanghai 200233, China

**Keywords:** 肺肿瘤, PI3K/Akt/mTOR, 信号转导, Lung neoplasms, PI3K/Akt/mTOR, Signal transduction

## Abstract

肺癌是目前世界上发病率和死亡率最高的恶性肿瘤之一，其中非小细胞肺癌（non-small cell lung cancer, NSCLC）占肺癌的75%-85%，确诊时多属中晚期，常规放、化疗效果欠佳，5年生存率仅为5%-10%。PI3K/Akt/ mTOR信号通路作为细胞内重要信号转导通路之一，通过影响下游多种效应分子的活化状态，与NSCLC的发生发展密切相关。本文综述了PI3K/Akt/mTOR信号通路的组成，其抑制凋亡、促进增殖的关键作用以及在NSCLC中的研究现状，以期为NSCLC的治疗寻找潜在的靶点。

## Abstract (en)

Lung cancer is one of the leading causes of cancer-related deaths worldwide and non-small cell lung cancer (NSCLC) accounts for about 75%-85% of all lung cancers. NSCLC often presents at stages too late for surgical intervention and traditional treatments including chemotherapy and radiotherapy are inadequate. The 5-year survival rate of NSCLC is only 5%-10%. PI3K/Akt/mTOR signaling pathway is an important intracellular signal transduction pathway. It plays an important role in cell apoptosis and survival by affecting the activity of downstream effector molecules, and it is closely associated with the development and progression of NSCLC. This article reviewed recent progress on the composition, anti-apoptosis and pro- proliferation of PI3K/Akt/mTOR signaling pathway, and discussed its potential targets to NSCLC therapy.

随着对肿瘤研究的不断进展，许多与肿瘤发生发展密切相关的信号通路被发现，PI3K/Akt/mTOR信号通路作为细胞内重要信号转导通路之一，通过影响下游众多效应分子的活化状态，控制着肿瘤发生发展中至关重要的细胞生物学过程，包括细胞凋亡、转录、翻译、代谢、血管新生以及细胞周期的调控。近年PI3K/Akt/ mTOR信号转导通路在非小细胞肺癌（non-small cell lung cancer, NSCLC）中的研究日益增多，本文对PI3K/Akt/ mTOR信号通路与NSCLC研究现状作一综述。

## 1 PI3K/Akt/mTOR信号转导通路简述

磷脂酰肌醇-3激酶（phosphatidylinositol 3-kinase, PI3K）是一种可催化磷脂酰肌醇D3位磷酸化的脂类激酶，在PI3K家族中，研究最广泛的是能被细胞表面受体所激活的Ⅰ型PI3K。IA型PI3K是由催化亚基p110和调节亚基p85所组成的二聚体蛋白，具有类脂激酶和蛋白激酶的双重活性^[1]^。随着各种生长因子作用于膜受体并使之活化，PI3K信号通路也因此而激活。PI3K通过两种方式激活：一种是与具有磷酸化酪氨酸残基的生长因子受体或连接蛋白相互作用，引起二聚体构象改变而被激活；另一种是通过Ras和p110直接结合导致PI3K的活化^[2]^。p110催化亚基继而磷酸化磷脂肌醇的肌糖环D3位点从而产生PI-3, 4, 5-P3（PIP3）。*Akt*基因是一个丝/苏氨酸蛋白激酶，又被称为蛋白激酶B，Akt是PI3K下游的作用靶点。PIP3结合到PDK1和Akt的PH结构域并使它们转位到细胞质膜，随后Akt催化结构域Thr308位点被PDK1磷酸化，而Ser473位点则被PDK2磷酸化，使Akt活化。哺乳动物雷帕霉素靶蛋白（mammalian target of repamycin, mTOR）是一种与PI3K/Akt通路相关的蛋白激酶，mTOR作为Akt的一个底物而被激活。PIP3作为第二信使在细胞中传递信号，介导PI3K通路的多种功能，包括细胞增殖和存活、细胞骨架重组、膜运输、细胞粘附和运动、血管再生及胰岛素作用^[3]^。*PTEN*是一种肿瘤抑制基因，位于人染色体10q23。PTEN可以调控PI3K信号途径。有证据^[4]^显示PTEN能使PIP3去磷酸化，因此作为负调控因子的PTEN的缺失会引起PI3K/Akt/mTOR通路的激活，从而导致肿瘤的发生，见图 1。

**1:**
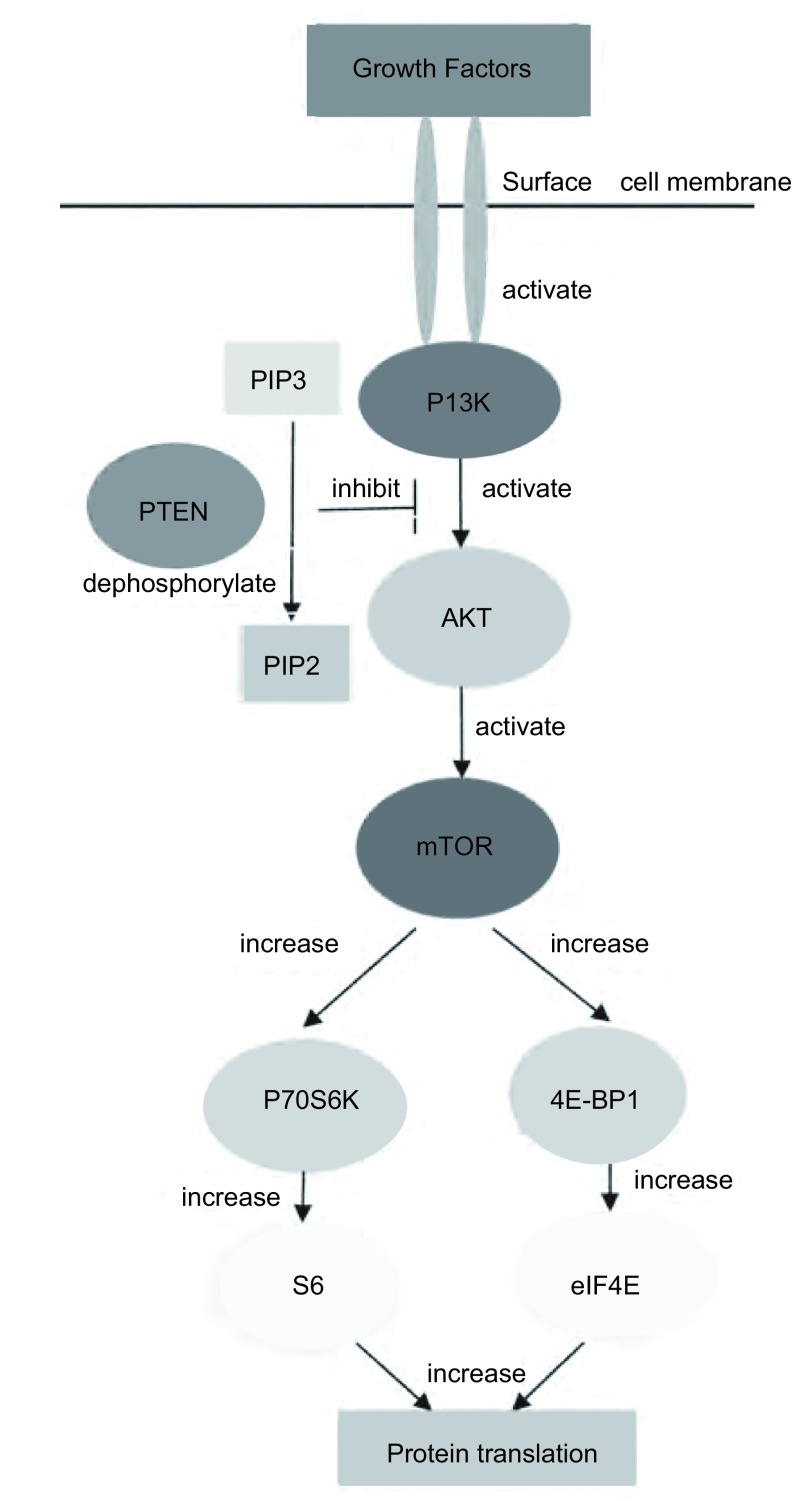
PI3K/Akt/mTOR轴。各种生长因子作用于跨膜受体并使其活化，PI3K信号通路也因此而激活。胞浆面PIP3浓度升高，作为第二信使与AKT的PH结构域结合分别使AKT Thr308和Ser473磷酸化导致AKT完全激活，继而磷酸化mTOR及其下游分子p70S6K、4E-BP1，下传生存信号。PTEN作为负调控因子，能使PIP3去磷酸将其转变为PIP2而降解，从而阻断AKT及其下游分子的有效活化。 PI3K/Akt/mTOR axis. PI3Ks are predominantly activated by growth factor receptor tyrosine kinases. Generation of PI (3, 4, 5) P3 within the cell membrane by class IA PI3Ks initiates a signaling cascade that activates AKT and mTOR. mTOR is a central control- ler of cell growth, cell division and protein translation, primarily through two distinct pathways: ribosomal p70 S6 kinase (p70S6K) and the eukaryotic translation initiation factor 4E (eIF4E) bind- ing proteins (4E-BPs). The reaction catalyzed by class IA PI3Ks is directly antagonized by PTEN which dephosphorylates the 3' position of PI (3, 4, 5) P3 to produce PI (4, 5) P2.

## 2 PI3K/Akt/mTOR信号转导通路在肿瘤的发生发展中的作用

### 2.1 抑制细胞凋亡

PI3K/Akt/mTOR信号通路的抗凋亡作用可能与下列几种机制相关：①调节Bcl-2家族成员的活性：目前已经发现的Bcl-2蛋白家族按功能可分为两类：一类是抑制凋亡的Bcl-2和Bcl-xL，另一类是促进凋亡的蛋白，如Bad、Bik、Bid等，细胞生存或凋亡的关键取决于促凋亡和抑凋亡蛋白之间的平衡。Akt可使Bad的Ser136/ Ser112残基磷酸化，磷酸化的Bad与Bcl-2或Bcl-xL解聚，Bad再与抗凋亡蛋白14-3-3结合，而游离的Bcl-2发挥抗凋亡作用^[5]^。此外，PI3K/Akt通路的激活可使Bax的Ser184残基磷酸化而失活，从而抑制细胞凋亡^[6]^；②caspase-9参与细胞凋亡的起始，caspase-3参与细胞凋亡的执行，活化的Akt可使caspase-9的Ser196和caspase-3磷酸化，阻止caspase-9和caspase-3的活化；③直接或间接影响转录因子家族如Forkhead、NF-κB、p53等；④Akt能通过磷酸化mTOR及其下游分子p70S6K、4E-BP1下传生存信号，抑制不依赖p53的细胞凋亡，促进细胞生存^[7]^；⑤Akt能抑制线粒体释放细胞色素C。

### 2.2 促进细胞增殖

参与细胞周期调控的主要分子有：细胞周期蛋白（cyclin）、细胞周期蛋白依赖激酶（cyclin dependent kinase, CDK）、CDK抑制蛋白（cyclin dependent kinase inhibitors, CKIs），它们组成一个网络系统，协调控制细胞周期。Akt通过对cyclin D1激酶糖原合成酶-3β（glucose syntase kinase-3β, GSK3β）的调节起到防止cyclin D1下调的作用。Akt直接磷酸化GSK3β并阻止激酶的活化，使cyclin D1累积^[8]^。此外，Akt还能抑制CKIs p27KIP1和p21CIP1/WAF1的表达并通过磷酸化的形式直接或间接调节p27和p21的激活。

### 2.3 促进血管生成

血管内皮生长因子（vascular endothelial growth factor, VEGF）是一类多功能生长因子，能特异性作用于血管内皮细胞，促进细胞增殖及血管形成。PI3K能传递整合素所介导的侵袭信号，与肿瘤的侵袭行为密切相关。PI3K通过与VEGF-2形成复合物经由PI3K/Akt通路的活化参与VEGF介导的内皮信号的传递，VEGFR-2与αVβ3复合物也以PI3K依赖的方式介导内皮细胞的粘附和迁移^[9]^。在结直肠肿瘤细胞中，肝细胞生长因子（hepatocyte growth factor, HGF）可通过MEK/ERK和PI3K/ Akt信号通路上调VEGF的表达^[10]^。PI3K/Akt通路的活化还可通过多种途径上调HIF-1α，促进VEGF的表达，使内皮细胞迁移形成新生血管，增加肿瘤细胞的血供。Akt可使内皮型一氧化氮合酶（endothelial NO synthase, eNOS）磷酸化来促进内皮生长因子诱导的内皮细胞迁移，导致新血管生成，还可活化转录因子NF-κB，NF-κB的活化不仅促成了癌细胞对血管壁的跨越，而且诱导了新生血管形成所需的趋化因子的基因转录。

## 3 PI3K/Akt/mTOR信号转导通路与NSCLC的发生发展

肺癌是目前世界上发病率和死亡率最高的恶性肿瘤，每年有超过100万人被确诊为肺癌^[11]^。发病率在多数国家呈增高趋势，其中NSCLC约占肺癌的75%-85%，大多数临床病例确诊时已属中晚期，失去了手术切除机会，而目前传统放、化疗效果欠佳，5年生存率仅为5%-10%。因此寻找新的更有效的治疗方法显得尤为重要。

### 3.1 NSCLC中PI3K/Akt/mTOR信号通路的激活在过去的

20年，PI3K信号通路在肿瘤发生、发展中的作用逐渐被证明。已有许多研究证明PI3K/Akt信号通路在NSCLC的生长中起重要作用。约有50%-70%的NSCLC中存在Akt的磷酸化，这表明IA型PI3K/Akt信号通路的激活在NSCLC中很常见。持续的PI3K的激活是上游信号分子基因的改变、PIK3CA的突变或扩增、PTEN的缺失或下游信号分子的活化等一系列因素作用的结果^[12]^。David等^[13]^采用免疫组化方法分析NSCLC标本发现磷酸化Akt（p-Akt）与肿瘤的侵袭和生存率的缩短相关。Marinov等^[14]^在51%的NSCLC患者样本和74%的NSCLC细胞系中发现持续的Akt激活和mTOR磷酸化。与多数研究结果不同，Shah等^[15]^发现p-Akt是一个有利的预后因素，当然，这与不同的肿瘤分期和淋巴结转移情况有关。统计学分析表明吸烟、肿瘤大小、淋巴结、远处转移、肿瘤分期、p-Akt和PTEN的缺失均与预后相关，而仅吸烟、肿瘤分期和PTEN表达是独立的预后因素^[4]^。有研究^[16]^报道，解除对PI3K/Akt/ mTOR信号转导通路的调控能促进肺癌的发生和发展，而应用PI3K抑制剂如LY294002能促进NSCLC细胞凋亡，增加化疗的敏感性。因此，PI3K信号通路在细胞增殖、生存、肿瘤的进展和化疗、放疗耐药等起重要作用。

### 3.2 NSCLC中PI3K/Akt/mTOR通路的基因突变

肿瘤通常是基因突变直接激活PI3K信号通路，尤以p110α（*PIK3C A*）的突变激活和PTEN缺失这两种情况最常见。尽管有证据^[17, 18]^显示在NSCLC中同样存在PIK3CA突变和PTEN表达缺失，然而却并不常见。在NSCLC中，仅有3%发生PIK3CA突变，而*PIK3C A*基因拷贝数目的增加却较为常见，这说明肺癌可能通过其它机制活化PI3K/Akt信号通路。在NSCLC中还发现两种IA型PI3K/ Akt/mTOR通路下游分子的突变。其一是Akt1（E17K）的pleckstrin同源结构域突变激活，其二是*LKB1*/*STK11*突变失活^[12]^。尽管体内*LKB1*突变在其它类型肿瘤中较少见，在NSCLC中却有较高的发生率，尤其常见于腺癌。该突变与吸烟史和*KRAS*突变相关。LKB1的缺失可能潜在增加*KRAS*的突变^[19]^。在NSCLC中由于PI3K/Akt的激活和LKB1的突变或失活，mTOR也被激活。在对mTOR的调控中可能有数个其它途径的聚合，包括LKB1/AMPK、MAPK/ RSK和Ⅲ型PI3K，不过在肺腺癌中IA型PI3K信号通路如何影响mTOR的活化仍有待进一步研究。

### 3.3 PI3K/Akt信号通路与NSCLC的转移

Grille等^[20]^研究表明，人鳞癌细胞系SCC15转染激活型Akt（myr-Akt）后丧失了鳞状上皮细胞的形态学特征，呈现出成纤维细胞样特点。说明Akt直接影响了上皮细胞的形态学特征、成瘤性及细胞的运动力和侵袭力，因此PI3K/Akt信号通路通过降低细胞间的粘附力来促进肿瘤细胞的转移。有研究^[21, 22]^发现，在肺癌细胞系A549中，转化生长因子-β1（transforming growth factor-β1, TGF-β1）和CC-趋化因子5（CC chemokine ligand 5, CCL5）通过刺激PI3K的p85α亚基和Akt的Ser473位点磷酸化分别上调β1整合蛋白和avβ3整合蛋白的表达，促进细胞转移。趋化因子受体-4（chemokine receptor 4, CXCR4）与mTOR调节的肿瘤转移密切相关，临床前研究表明，NSCLC A549细胞和H157细胞在缺氧环境中培养，导致细胞表面CXCR4明显上调，并且发现这两种肿瘤细胞迁移能力增强。缺氧和EGR促发的PI3K/Akt活化协同作用使CXCR4表达增加提示mTOR通路在CXCR4上调中的作用。此外，通过PI3K/ Akt/mTOR途径可以上调一些基质金属蛋白酶的表达来促进肿瘤细胞的转移，Zhang等^[23]^发现高侵袭性Lewis肺癌细胞亚系H-59表达MT1-MMP，PI3K抑制剂和mTOR抑制剂Rapamycin以及Akt的显性负突变体和PTEN的过度表达均能阻碍细胞MT1-MMP的表达，减少细胞的侵袭性。

## 4 以PI3K/Akt/mTOR信号转导通路为靶点的药物研究

随着对PI3K/Akt/mTOR通路在肿瘤发病中的作用的逐步认识，以PI3K/Akt/mTOR信号轴为靶点的新药研发越来越受到重视。目前已陆续开发出了一系列以该通路为靶标的特异性药物。这些药物可直接抑制PI3K/Akt/ mTOR通路中过度活化的信号转导分子，从而对致病环节起到阻断作用。其中具有代表性的几类药物介绍如下。

### 4.1 PI3K抑制剂

Wortmannin和LY294002作为第一代PI3K抑制剂，能特异性抑制PI3K的p110亚基的催化活性，阻断PI3K/Akt通路的活化。将Wortmannin或LY294002与化疗药物联合使用能够有效地增加化疗药物的作用并降低毒性，这表明PI3K抑制剂与传统化疗药物的联用为已对传统化疗药物产生耐药的肿瘤患者提供了更好的选择^[24]^。尽管Wortmannin和LY294002有很好的抗肿瘤活性，但由于毒性较强，限制了它们在临床上的使用。PX-866和PWT-458是近年来新发现的Wortmannin的衍生物，具有高效的PI3K抑制作用。在肺癌模型中PX-866与顺铂或放疗联用能增强抗肿瘤作用^[25]^。PW T-458（pegylated-17- hydroxywortmannin）的活性成分是17-HWT，实验证明静脉注射PWT-458后，裸鼠异体移植瘤中磷酸化的Akt完全消失，且在裸鼠NSCLC A549移植瘤模型中具有抗肿瘤活性，此外，PWT-458还能增强紫杉醇的抗肿瘤疗效^[26]^。理论上，PI3K抑制剂可以避免由于抑制mTOR而产生Akt的反馈性激活。所以对于非影响ATP结合类型抑制剂的研发会产生更加高效、特异、低毒的抑制剂^[27]^。

### 4.2 Akt抑制剂

较早发现的Akt抑制剂有celecoxib（塞来昔布）及其衍生物OSU-03012和OSU-03013。celecoxib是一种COX-2的抑制剂，能抑制PDK的作用而阻止Akt磷酸化。多项临床试验^[28, 29]^显示单用celecoxib^[30]^或是与docetaxel（多西他赛）或zoledronate（唑来磷酸）联用均显现出较好的治疗效果。perifosine是一种基于脂的Akt抑制剂，通过抑制Akt的膜转位，降低Akt的活性，抑制多种肿瘤细胞的生长^[27]^。临床Ⅱ期研究正评价perifosine对于复发性胰腺癌、前列腺癌、头颈癌、肺癌的疗效。抑制Akt的优势在于可以控制众多的下游信号传递，因为Akt下游底物并未被完全了解，这就造成直接抑制下游底物的困难。因此抑制Akt能够更加有效发挥作用，但其代价是更大的潜在毒性^[31]^。

### 4.3 mTOR抑制剂

有研究^[32]^显示，在74%的NSCLC中发现磷酸化或活化的mTO R，因此mTO R成为又一个NSCLC治疗的靶标。目前，数项肿瘤临床试验的作用评估mTOR抑制剂雷帕霉素及其衍生物（CCI-779, RAD001, AP23573）。mTOR抑制剂在肿瘤治疗中显示了很好的耐受性，皮肤反应、口腔炎、骨髓压迫等常见的副反应通常是短暂和可逆的。已有多种证据显示mTOR抑制剂的抗肿瘤作用能缓解病情或延长稳定期，其中也包括NSCLC。这些令人鼓舞的初期临床试验数据将推动mTOR抑制剂的进一步研究^[33]^。雷帕霉素的抗增殖作用可能是由于cyclins尤其是cyclin D的减少抑制CDK的活化，以及CKIs p21CIP1和p27KIP1的增加共同阻止G1期的进程。同时，雷帕霉素还具有促凋亡和抑制血管再生的作用。现已证实雷帕霉素能有效抑制人NSCLC细胞生长，而它与多西他赛联用对抑制肺癌细胞生长具有协同作用^[34]^。Konstantinidou等^[35]^研究发现，对PI3K/mTOR有双重阻断作用的BEZ235联合放疗能使携带K-R A S的NSCLC患者受益。这表明在肺癌治疗中，mTOR抑制剂与传统放、化疗联用或许会显现出更好的疗效。

值得注意的是，表皮生长因子受体酪氨酸激酶抑制剂（epidermal growth factor receptor tyrosine kinase inhibitor, EGFR-TKIs）作为目前成功应用于NSCLC治疗的小分子靶向药物，许多患者对EGFR-TKIs却无反应或产生耐药，其中一个重要的机制是由于抑制EGFR活性的信号通路下游发生了改变，例如PI3K通路的持续激活。已有研究^[36]^表明，HKI-272联合雷帕霉素能使小鼠的肿瘤体积减少（72±4.5）%，对后天获得厄洛替尼耐药的NSCLC患者可能产生敏感的反应。此外，有研究^[37]^发现用吉非替尼治疗的NSCLC患者中p-A kt阳性患者在治疗反应、疾病控制和生存时间方面好于p-Akt阴性患者。除了常规*EGFR*突变检测外，p-A kt能辅助预测患者对吉非替尼的敏感性^[38]^。而Pu等^[39]^通过对PI3K/PTEN/AKT/mTOR通路5个核心基因单核苷酸多态性（single necleotide polymorphism, SNP）标签来识别SNPs相关的毒性和疾病进展，发现PI3K/ PTEN/Akt/mTOR通路的遗传变异能预测接受铂类化疗药的NSCLC患者的药物毒性和肿瘤的远处进展情况。由此可见，针对不同信号通路多靶点治疗可能是NSCLC治疗更好的策略，而PI3K/Akt/mTOR信号环中反馈和串音的存在也说明要获得最佳的治疗效果可能需要多水平和（或）多通路同时抑制^[12]^。

## 5 结语

PI3K/Akt/mTOR信号通路与NSCLC的发生发展、治疗及转归密切相关，但目前仍有许多问题不甚明了。同时，各条信号通路之间相互交叉形成了复杂的信号网，例如PI3K/Akt信号通路与Ras/丝裂原活化蛋白激酶信号通路、生长因子信号通路相互联系、相互影响，可能共同对NSCLC的发生起重要作用。进一步研究该通路的调节及与其它通路之间的交联，从而深入了解该通路在NSCLC中的作用，将有助寻找新的治疗靶点。
